# Identification and Transcriptome Analysis of Genes Related to Membrane Lipid Regulation in Sweet Sorghum under Salt Stress

**DOI:** 10.3390/ijms23105465

**Published:** 2022-05-13

**Authors:** Fenghui Wu, Zengting Chen, Fangning Zhang, Hongxiang Zheng, Simin Li, Yinping Gao, Jie Yang, Na Sui

**Affiliations:** Shandong Provincial Key Laboratory of Plant Stress, College of Life Sciences, Shandong Normal University, Jinan 250014, China; wufhsdnu@163.com (F.W.); 15653771318@163.com (Z.C.); zhangfangning423@126.com (F.Z.); zhenghongxiang0208@gmail.com (H.Z.); lsm961204@126.com (S.L.); gyp970326@163.com (Y.G.); yangjie135792020@163.com (J.Y.)

**Keywords:** transcriptomic profile, salt stress, membrane lipid metabolism, enzyme regulation, sweet sorghum

## Abstract

Sweet sorghum has strong stress resistance and is considered a promising energy crop. In the present study, the effects of salt on the membrane lipid metabolism of two sweet sorghum inbred lines (salt-tolerant M-81E and salt-sensitive Roma) were analyzed. After treatment with 150 mM NaCl, higher levels of fresh weight and chlorophyll fluorescence, as well as lower levels of malondialdehyde (MDA) were found in salt-tolerant M-81E. Concomitantly, 702 and 1339 differentially expression genes (DEGs) in M-81E and Roma were identified in response to salt stress. We determined that most DEGs were related to glycerophospholipid metabolism, glycerolipid metabolism, and other membrane lipid metabolisms. Under NaCl treatment, the expression of the membrane-associated phospholipase A1 was down-regulated at the transcriptional level, along with an increased content of phosphatidylcholine (PC) in both cultivars. The inhibition of triacylglycerol (TAG) mobilization in M-81E delayed salt-induced leaf senescence. Furthermore, enhanced levels of glycerol-3-phosphate acyltransferase (GPAT) expression contributed to improved salt resistance in M-81E. The results of this study demonstrate membrane the role of lipid regulation in mediating salt-defensive responses in sweet sorghum and expand our understanding of the relationship between changes in membrane lipid content and salt resistance.

## 1. Introduction

Salinity is one of the main unfavorable environmental factors that restrict plant growth and has led to a decline in global crop production [1,2,3,4,5,6]. The injurious effect of salinity stress on plant growth takes place in two major ways: osmotic stress and ionic toxicity [7,8,9]. High salt ion concentrations cause disturbances in photosynthesis, cellular metabolism, and nutritional balance [10]. In addition, a large number of studies have shown that salt stress is the causal factor of modifications in lipid bilayer composition and membrane permeability [11,12,13]. Such modifications can directly induce the alteration of membrane fluidity and H^+^-ATPase activity, influencing the passive influx of potentially toxic ions such as Na^+^ and Cl^−^ [13,14]. As a biological barrier, the cell membrane can protect the cells and organelles against various stresses, which is an important mechanism that produces salt tolerance in plants [15,16].

Plant membrane lipids are divided into three categories: sphingolipids, sterols, and glycerolipids [17]. Sphingolipids have a ceramide backbone, which affects the integrity and permeability of the cell membrane [18]. Sterols mainly have structural and regulatory effects. Changes in membrane sterol composition might be important for protecting membranes from environmental stress [19]. Glycerolipids are the principal constituents of biological membranes and have been involved in the dynamic signaling processes associated with aspects of plant development and resistance to abiotic stresses [20]. According to their molecular structure, glycerides can be divided into lipids formed by cylindrical bilayers and lipids formed by conical non-bilayers [21]. Generally, bilayer lipids ensure membrane stability, while non-bilayer lipids are important for mediating protein-lipid interactions and increasing the morphological plasticity of lipid bilayers [22,23].

The plasma membrane might be the primary site of salinity injury [14,24]. Under salt stress, cell membrane lipids will undergo a degreasing reaction or membrane lipid peroxidation, thereby damaging membrane proteins and membrane lipid structures [25,26,27,28]. Changes in plasma membrane lipids might help to maintain the stability and integrity of the membrane’s structure as a stress-adaptation mechanism [12]. Studies have proposed that salinity decreases the unsaturated fatty acid content of lipids. However, the unsaturated fatty acid content of lipids has been found to be higher in a salt-tolerant maize cultivar compared with in a salt-sensitive cultivar [29]. Lipid reprogramming along with changes in lipid content, composition, and saturation levels could regulate membrane stabilization and salt tolerance. Sweet sorghum (*Sorghum bicolor (L.)* Moench) has strong resistance to abiotic stress [30,31,32,33]. However, the mechanisms of salt tolerance involving lipid metabolism are not clear.

Our previous research revealed that the differences in salt tolerance between the salt-tolerant sweet sorghum line (M-81E) and the salt-sensitive line (Roma) are linked to genes involved in diverse signaling pathways [34,35]. Since membrane composition is fundamental to the adaptation of plants to salt stress, the present study involved performing high-throughput Illumina RNA sequencing (RNA-seq) analysis to identify membrane lipid metabolic changes and investigate the regulation of lipid remodeling in two sorghum genotypes with different salt tolerances under salt stress. The results of this study will provide new insight into the potential role of membrane lipids in the regulatory networks underlying the mechanisms of salt tolerance in sweet sorghum.

## 2. Results

### 2.1. Effects of Salt Stress on Plants

**Figure 1:**
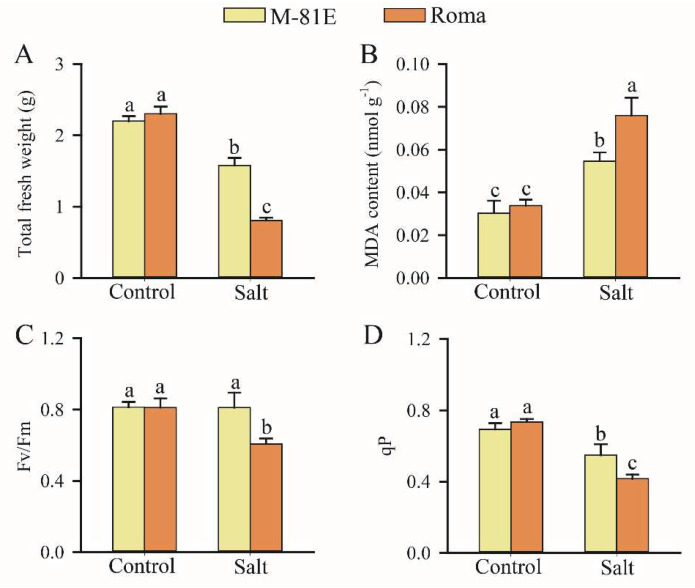
Effect of salt stress on total fresh weight (**A**), MDA content (**B**), the maximum quantum yield of PSII (**C**), and qP (**D**) of salt-tolerant (M-81E) and salt-sensitive (Roma) genotypes. Each value represents the mean of 6 replicates ± SD. Different letters indicate a significant difference (*p* < 0.05) between the control and the treatment.

Our results showed that salt stress produced significantly lower fresh weights for both cultivars when compared with non-saline conditions. However, the M-81E cultivar had a higher fresh weight under salt conditions than the Roma cultivar (Figure 1A). Malondialdehyde (MDA) content was increased by salt stress, but the increase in Roma under salt stress was higher than that in M-81E (Figure 1B). The maximal quantum yield of Photosystem II (Fv/Fm) for M-81E leaves showed no significant difference between salt stress and control conditions, but in Roma the ratio of Fv/Fm significantly decreased in response to salt stress (Figure 1C). Under the salt environment, the photochemical quenching coefficient (qP) of all lines was markedly reduced and Roma had lower qP values when compared with M-81E (Figure 1D).

### 2.2. Identification of Differential Expression Genes (DEGs) Involved in Salt Stress

**Figure 2:**
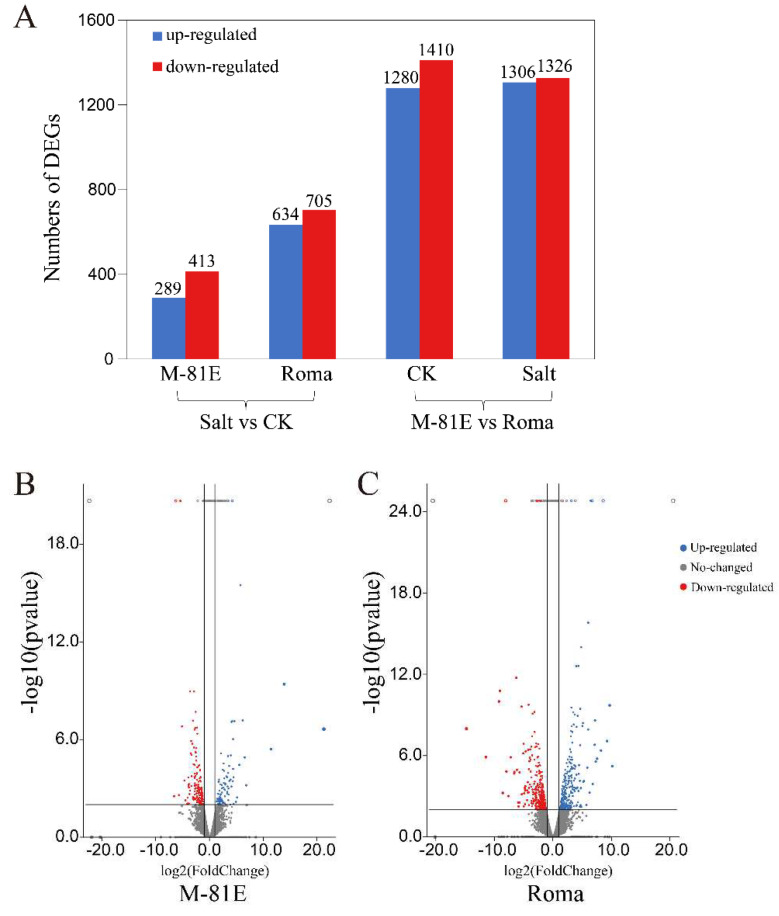
Numbers of differentially expression genes (DEGs) of different genotypes when affected by salt stress. (**A**) DEG distribution of the two treatment groups under normal soil or salt stress conditions; (**B**) volcano plot showing DEGs for M-81E+salt and M-81E; (**C**) volcano plot showing DEGs for Roma+salt and Roma.

Under control conditions, we identified 2690 genes differentially expressed in the M-81E group when compared with the Roma group. Of these, 2632 were DEGs in the presence of salt (Figure 2A). In the gene expression profiles of M-81E, 702 DEGs were identified when comparing the control with the salt treatment. Of these genes, 289 were up-regulated (Figure 2B). In the Roma cultivar, 1339 genes were differentially expressed between control and salt-stressed plants, of which 634 genes were up-regulated under salt stress (Figure 2C). DEGs related to membrane lipid metabolism were selected for further analysis.

### 2.3. Functional Classification by Gene Ontology (GO)

**Figure 3:**
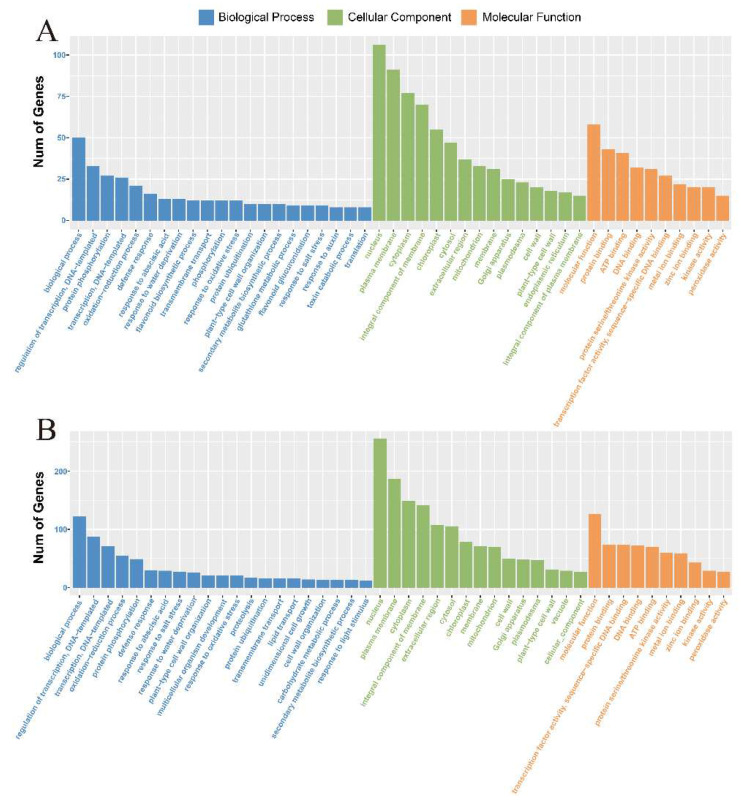
Functional annotation of assembled sequences based on gene ontology categorization in M-81E (**A**) and Roma (**B**). Results are summarized under three main functional classes: biological processes, molecular functions, and cellular components.

DEGs were evaluated using GO analysis to identify their functional information [36]. These DEGs could be categorized into three main functional classes—biological processes, molecular functions, and cellular components—using GO categories. In M-81E and Roma, 1344 and 2788 transcript sequences, respectively, were assigned to 50 s-level GO categories. These were summarized using the functional classes as follows: 15 cell components, 10 molecular functions, and 25 biological processes. For M-81E and Roma, “nucleus” was the main term of the cell component category. In terms of molecular function, “molecular_function” and “protein binding” were significantly richer terms. In terms of biological process categories, “biological_process” and “regulation of transcription, DNA-templated” were significantly enriched terms (Figure 3A,B).

### 2.4. Functional Classification by KEGG

**Figure 4:**
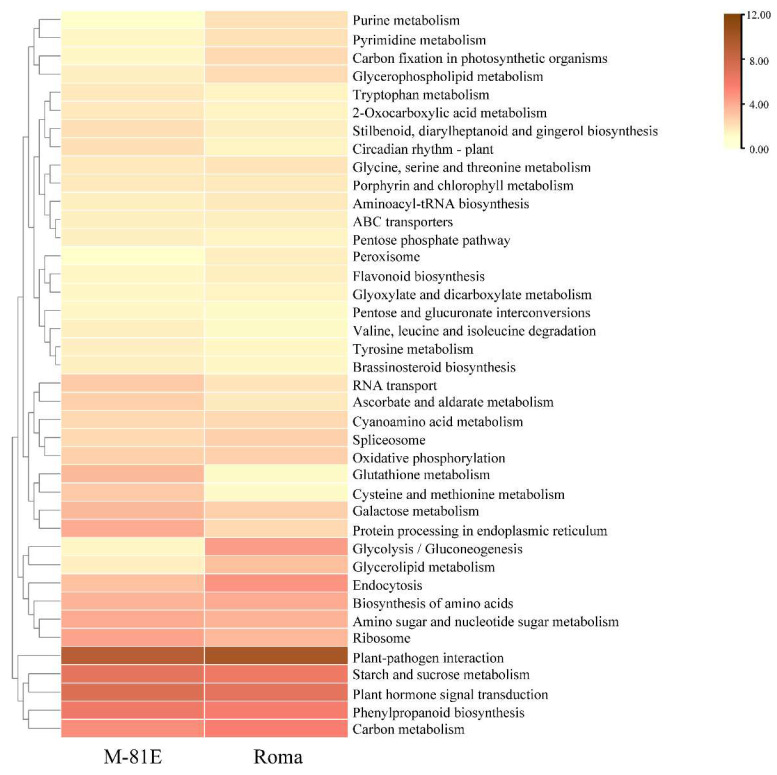
The heat map display of DEGs assigned to different KEGG pathways.

A Kyoto Encyclopedia of Genes and Genomes (KEGG) analysis was also performed on the DEGs to explore metabolic pathways in the transcriptome of sweet sorghum [37]. Through the analysis of DEGs, it was found that the KEGG pathway related to membrane lipids was mainly concentrated on the glycerolipid metabolism and glycerophospholipid metabolism pathways, which indicated that salt stress regulated membrane lipid metabolism mainly by affecting glycerolipid metabolism and glycerophospholipid metabolism (Figure 4).

### 2.5. DEGs Related to Membrane Lipid Metabolism under Salt Stress

**Figure 5:**
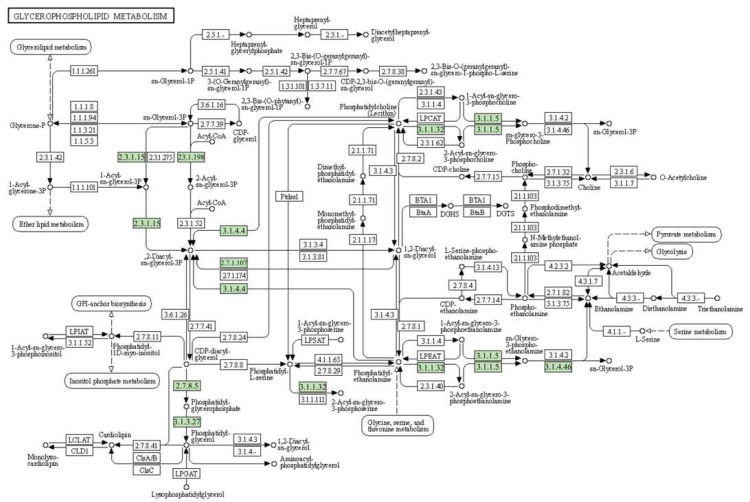
Overview of the glycerophospholipid metabolism pathways in sweet sorghum. Through the genes in the light green frames, we can see which pathways were involved among the key DEGs in both sweet sorghum genotypes.

Salt stress can cause membrane lipid changes (including in membrane lipid content and signal lipid activity) and ultimately change the biological characteristics of membrane lipids [38]. Changes in plant membrane lipids cause lipid biosynthesis and metabolic enzyme regulation [39]. In this study, 25 genes were mapped to two pathways related to membrane lipid metabolism, of which 11 were mapped to glycerolipid metabolism while the other 14 were mapped to glycerophospholipid metabolism (Figure 5 and Figure S1). It can be seen in Table 1 that in the glycerol membrane lipid metabolism pathway, M-81E had six DEGs encoding three enzymes and Roma had five genes encoding four enzymes. In the glycerophospholipid metabolism pathway, 6 DEGs in M-81E encoded 5 enzymes and 10 DEGs in Roma encoded 7 enzymes.

From the results in Table 1, we analyzed the pathway of glycerolipid metabolism. In M-81E, the DEGs (SORBI_3010G270700 and SORBI_3002G067000) that encode the phospholipid diacylglycerol acyltransferase (EC:2.3.1.158) were significantly down-regulated, while in Roma the DEGs (SORBI_3010G001700 and SORBI_3004G286700) that encode the phospholipid diacylglycerol acyltransferase were significantly up-regulated. In M-81E, the DEGs that encode glycerol-3-phosphate O-acyltransferase (EC:2.3.1.15) and alpha-galactosidase (EC:3.2.1.22) were significantly up-regulated. In Roma, the DEG that encodes 1-acyl-sn-glycerol-3-phosphate acyltransferase (EC:2.3.1.51) was significantly up-regulated, while the DEGs that encode 1, 2-diacylglycerol 3-beta-galactosyltransferase (EC:2.4.1.46) and acylglycerol lipase (EC:3.1.1.23) were significantly down-regulated. Through DEG analysis of the glycerophospholipid metabolism pathway, phospholipase D1/2 (EC:3.1.4.4), which is encoded by SORBI_3005G222500, was found to be significantly down-regulated in M-81E and Roma, and glycerophosphodiester phosphodiesterase (EC:3.1.4.46), which is encoded by SORBI_3004G157300, was also found to be significantly down-regulated in M-81E and Roma.

### 2.6. qRT-PCR Validation of DEGs

**Figure 6:**
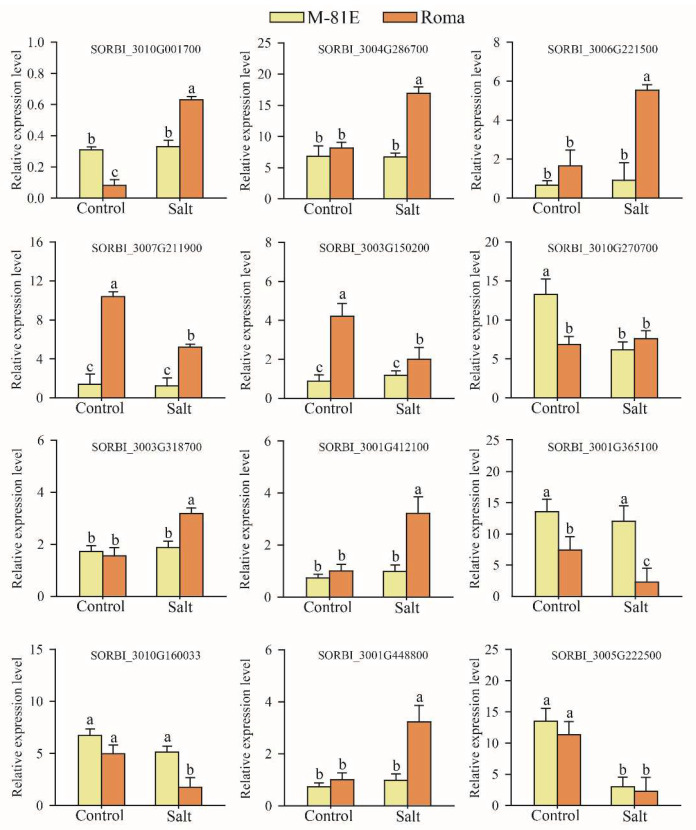
Verification of expression patterns of selected genes.

The analysis of the DEGs and the qRT-PCR verified that the fragments per kb per million (FPKM) method could be used to calculate gene expression levels. To verify the RNA-seq data, 12 DEGs related to membrane lipid regulation were selected for quantitative real-time PCR analysis. These included genes related to glycerolipid regulation and glycerophospholipid regulation. Using qRT-PCR analysis, the expression of these genes was found to show a similar pattern to that of genes produced using FPKM values sequenced under corresponding treatments (Figure 6). These results indicated that the RNA-seq data were reliable.

## 3. Discussion

In the present investigation, the two sweet sorghum inbred lines showed different physiological responses to salt stress. Upon exposure to salt stress, total fresh weight was found to be significantly higher in the salt-tolerant M-81E than in the salt-sensitive Roma, which could help M-81E to perform physio-biochemical processes more efficiently under the effects of water deficit. Lipid peroxidation measured as the amount of MDA is considered to be an indicator of oxidative damage from stress [40,41]. MDA content was increased by NaCl treatments in both genotypes. However, salt-tolerant M-81E had less MDA content than Roma. From these findings, it can be inferred that a higher membrane stability index and water retention capacity might have imparted salt stress tolerance to M-81E.

It can be seen from the KEGG pathway of glycerophospholipid metabolism that phosphatidylcholine (PC) is an important intermediate phospholipid and is the main component of the membrane. Lipid metabolism has been found to contribute significantly to the ability of plants to survive under abiotic stresses [42]. Furthermore, PC levels have been found to significantly increase in callus cultures of the halophyte *Spartina patens* and the roots of salt-tolerant Plantago cultivars [43,44]. The addition of choline, a key substrate for PC biosynthesis, resulted in enhanced salt resistance in wheat, which was evidence for the positive regulatory role of PC [45]. In our study, the DEGs encoding phospholipase A1 that were responsible for catalyzing the acidolysis of PC were both significantly down-regulated in Roma and M-81E. Increased PC accumulation seems to be an indispensable adaptive response of plants to osmotic stress caused by salt damage.

**Figure 7:**
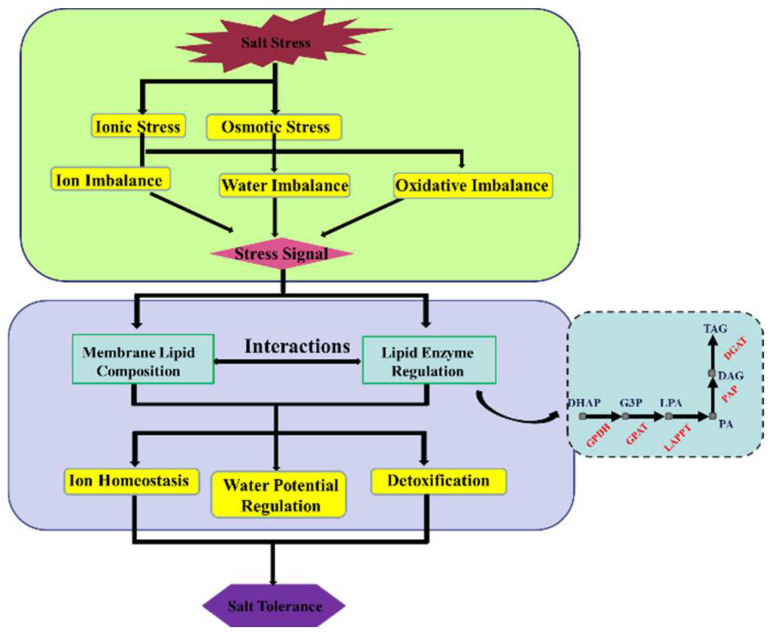
Salt stress mechanisms and membrane responsive regulations of membranes in plant cells. When a plant is subjected to salt stress, it will induce ion stress and osmotic stress, which will produce ionic, water, and oxidative imbalance signals. These, in turn, will induce membrane lipid regulation through metabolic pathways for glycerophospholipids and glycerol membrane lipids.

Glycerolipids are the most abundant lipids in higher plants. They include phospholipids, glycolipids, oils, and extracellular lipids, which are widely involved in different biological processes [46]. The synthesis of glycerolipid catalyzes the lipid acylation of sn-glycerol-3-phosphate (G3P) in glycerol-3-phosphate acyltransferase (GPAT) to form lysophosphatidic acid (LPA), and this is followed by the formation of phosphatidic acid PA under the catalysis of 1-acyl-sn-glycerol-3-phosphate acyltransferase (LPAAT) [47,48]. The PA produced in eukaryotic cells can be dephosphorylated in the endoplasmic reticulum to produce DAG, and then be catalyzed by diacylglycerol acyltransferase (DGAT) to form triacylglycerol (TAG) [49] (Figure 7). The reaction process that occurs in the endoplasmic reticulum through GPAT, LPAAT, and DGAT successively catalyzes the sn-1, sn-2, and sn-3 lipid acylation of G3P to form TAG, also known as the Kennedy pathway [49,50]. In the process of glycerolipid metabolism, SORBI_3003G360700 encoding GPAT was upregulated in M-81E in this study. The overexpression of SsGPAT in *Arabidopsis* can prevent chlorophyll content from decreasing and help maintain the level of unsaturated fatty acids. At the same time, it reduces the photoinhibition of PSII and PSI, protects the photosynthesis equipment, and maintains the membrane’s resistance to salt stress. In contrast, all these factors have been found to be reduced in SsGPAT t-DNA insertion mutants under salt conditions [51]. The expression of GPAT involved in photosynthetic activities possibly explains the higher levels of chlorophyll fluorescence that were obvious in M-81E subjected to salt stress.

In higher plants and microalgae, TAG biosynthesis occurs through acyl-CoA-dependent or acyl-CoA-dependent pathways. Alternatively, TAG can be formed through the catalysis of membrane-bound diacylglycerol acyltransferase (PDAT) through an acyl-CoA-independent pathway. In addition to having a strong relationship with the accumulation of lipids in tissues, PDAT also plays a role in lipid metabolism during plant seed germination and leaf senescence [52]. SORBI_3006G221500, which encodes LAPPT, and two DEGs that encode PDAT (SORBI_3010G001700, SORBI_3004G286700), were significantly up-regulated in Roma, and two other DEGs encoding PDAT (SORBI_3010G270700, SORBI_3002G067000) were significantly down-regulated in M-81E. These DEGs expressed enhanced TAG abundance in salt-sensitive Roma leaves but reduced abundance in salt-tolerant M-81E. These findings are consistent with the results of Silva et al. (2003), who found that leaves of the salt-sensitive sweet potato cultivar Xu 32 maintained a higher abundance of TAG under saline conditions [53]. Elevated TAG in vegetative tissues can act as an energy storage reservoir during stressful periods [54,55]. However, a massive accumulation of TAG is frequently observed in the senescing leaves of plants, which reflects the low salt resistance of plants [56,57]. The down-regulation of TAG is considered to be a strategy adopted by salt-tolerant plants to ameliorate the adverse effects of salt stress [58].

## 4. Materials and Methods

### 4.1. Plant Material and Salt Treatment

According to our previous research, M-81E was deemed to be a salt-tolerant inbred line, while Roma was considered to be a salt-sensitive inbred line [59]. Sweet sorghum M-81E and Roma seeds were washed with flowing water for at least 8 h, then planted in plastic basins and irrigated with running water. The germinated seeds were irrigated with a half-strength Hoagland’s nutrient solution containing 2500 μM Ca(NO_3_)_2_·4H_2_O, 2500 μM KNO_3_, 1000 μM MgSO_4_·7H_2_O, 500 μM KH_2_PO_4_, 23.125 μM H_3_BO_3_, 4.57 μM MnCl_2_·H_2_O, 0.38 μM ZnSO_4_·H_2_O, 0.16 μM CuSO_4_·5H_2_O, 0.055 μM H_2_MoO_4_·H_2_O, 10.02 μM FeSO_4_·7H_2_O, and 10.005 μM Na_2_EDTA·2H_2_O [60]. The seedlings were placed in an artificial intelligence incubator and cultivated at 28 ± 3 °C (day/night), with an illumination intensity of 600 µmol m^−2^ s^−1^ and 70% relative humidity (15 h photoperiod). Previous experimental investigation showed that 150 mM NaCl is a suitable concentration [59]. When sweet sorghum had grown to the three-leaf stage, seedlings were treated with a nutrient solution of between 0 and 150 mM NaCl. The NaCl content was increased by 50 mM every 12 h until it reached 150 mM. After 48 h of NaCl treatment, the total fresh weight and chlorophyll fluorescence profiles of each sample were measured immediately. The roots and leaves of three-leaf seedlings were then sampled and used for subsequent analyses.

### 4.2. Determination of MDA and Chlorophyll Fluorescence

The MDA content of the leaves was determined using the thiobarbituric acid reaction described by Heath and Packer [61]. After the leaf was darkened for 20–30 min, fluorescence levels (Fo and Fm) were measured using an FMS-2 portable modulated fluorometer (Hansatech, King’s Lynn, United Kingdom). A qP coefficient was then calculated using the saturation pulse method.

### 4.3. Construction and High-Throughput Sequencing of Transcriptome Libraries

Trizol reagent (Invitrogen, California, USA) was used to extract total RNA following the steps provided by the manufacturer. Total RNA quantity and purity were analyzed using a Bioanalyzer 2100 and an RNA 6000 Nano LabChip Kit (Agilent, Santa Clara, CA, USA). Ribosomal RNA was depleted using approximately 10 ug of total RNA representing a specific adipose type. After purification, poly(A)- or poly(A) + RNA fractions were broken into small pieces. Then the cleaved RNA fragments were reversely transcribed to create the final cDNA library, with an average insertion length of 300 bp (±50 bp) for the paired-end libraries. We used Illumina Hiseq 4000 (Lc-Bio, Hangzhou, China) to perform paired-end sequencing following the vendor’s recommendation.

### 4.4. Transcriptome Assembly and Identification

Cutadapt was used to obtain clean reads [62]. The quality of sequenced genomes of sweet sorghum was verified using the FastQC, Bowti2, and TopHat2 methods. We then combined all transcripts from the sweet sorghum samples and used Perl scripts to rebuild an overall transcriptome [63,64]. StringTie and Ballgown were used to assess the expression levels of all transcripts [65,66].

### 4.5. Identification and Functional Annotation of DEGs

StringTie was used to assess mRNA expression levels by calculating FPKM. Differential expression of mRNAs was screened using the R package Ballgown with|log2(fold change)| > 1 and with statistical significance (*p*-value < 0.05) [64,65]. To obtain highly similar sequences, single-gene sequences were aligned with those in the NR, KEGG, and Swiss-Prot databases [37,67,68]. We used the BLAST tool to obtain COG, and BLASTN to obtain the nucleotide database NT [69]. The KEGG database can analyze gene products. The online KEGG web server was used to distribute KEGG pathways to the assembly sequence.

### 4.6. Quantitative Real-Time PCR Analysis

Real-time fluorescence quantitative PCR was used to verify RNA-seq results. The Beacon Designer software (Palo Alto, California, USA) was used to design the primers of 12 genes (Table S1). RNA samples of M-81E and Roma plants were prepared using a total RNA extraction kit (Huayueyang, Beijing, China) [59]. We used tap root tissue to isolate total RNA and a Nanodrop-ND-1000 spectrophotometer (Wilmington, DE, USA) to quantify RNA. The housekeeping gene β-actin (GenBank ID: X79378) from Streptococcus bicolor was used as an internal standard. We carried out sample preparation according to the manufacturer’s instructions. A real-time quantitative PCR instrument (Roche Diagnostics, Meylan, France) was used to perform real-time PCR. The relative expression level of each gene was calculated using the 2^−^^△△Ct^ method [70].

### 4.7. Statistical Analysis

All data were evaluated using one-way ANOVAs in SPSS, version 25.0 (IBM, Armonk, New York, NY, USA). A *p*-value of <0·05 was taken as statistically significant.

## 5. Conclusions

This study demonstrates the detailed changes in the membrane lipid metabolism of two sweet sorghum cultivars when subjected to salt stress. Key genes involved in glycerolipid metabolism or glycerophospholipid metabolism were identified by comparing control and salt-stressed plants. The adjustment of lipid metabolism in response to salt stress could contribute to an increase in salt tolerance, as displayed by the improved expression profile of GPAT, coupled with TAG mobilization performed cooperatively in mediating salt-defensive responses in sweet sorghum leaves. The results of this study provide new insight into the potential role of the membrane lipid regulatory networks underlying the mechanisms of salt tolerance in sweet sorghum.

## Figures and Tables

**Table 1:** DEGs mapped to KEGG pathways related to lipid metabolism.

Gene ID	Anotation		M-81E			Roma	
		Log_2_FC	Regulated	*p*-Value	Log_2_FC	Regulated	*p*-Value
(A) Glycerolipid Metabolism							
SORBI_3010G001700	phospholipid:diacylglycerol acyltransferase	-	-	-	2.26	up	0.02
SORBI_3004G286700	phospholipid:diacylglycerol acyltransferase	-	-	-	1.09	up	0.04
SORBI_3006G221500	1-acyl-sn-glycerol-3-phosphate acyltransferase(LAPPT)	-	-	-	1.43	up	0.04
SORBI_3007G211900	1,2-diacylglycerol 3-beta-galactosyltransferase	-	-	-	−1.65	down	0
SORBI_3003G150200	acylglycerol lipase	-	-	-	−1.41	down	0.02
SORBI_3010G270700	phospholipid:diacylglycerol acyltransferase (PDAT)	−1.18	down	0.02	-	-	-
SORBI_3002G067000	phospholipid:diacylglycerol acyltransferase (PDAT)	−1.05	down	0.02	-	-	-
SORBI_3003G360700	glycerol-3-phosphate O-acyltransferase	1.65	up	0.02	-	-	-
SORBI_3002G417800	alpha-galactosidase	1.25	up	0.01	-	-	-
SORBI_3001G208100	alpha-galactosidase	1.71	up	0	-	-	-
SORBI_3001G208200	alpha-galactosidase	2.52	up	0	-	-	-
(B) Glycerophospholipid Metabolism							
SORBI_3006G221500	1-acyl-sn-glycerol-3-phosphate acyltransferase				1.43	up	0.04
SORBI_3003G318700	diacylglycerol kinase (ATP)				1.14	up	0.04
SORBI_3001G412100	CDP-diacylglycerol-glycerol-3-phosphate 3-phosphatidyltransferase				1.53	up	0.01
SORBI_3001G365100	phospholipase A1				−2.56	down	0
SORBI_3010G160033	phospholipase A1				−1.61	down	0
SORBI_3001G448800	lysophospholipase II				1.61	up	0.03
SORBI_3005G222500	phospholipase D1/2	−1.15	down	0.02	−1.88	down	0
SORBI_3008G183400	phospholipase D1/2				−1.73	down	0.01
SORBI_3004G157300	glycerophosphodiester phosphodiesterase	−1.04	down	0.01	−1.68	down	0
SORBI_3007G190700	glycerophosphodiester phosphodiesterase				−1.09	down	0.03
SORBI_3010G160000	phospholipase A1	−3.86	down	0.01			
SORBI_3009G014600	phospholipase A1	−1.35	down	0.01			
SORBI_3003G360700	glycerol-3-phosphate O-acyltransferase (GPAT)	1.77	up	0.03			

## Data Availability

The original contributions presented in the study are included in the article; further inquiries can be directed to the corresponding authors.

## Supplementary Materials

The following supporting information can be downloaded at: https://www.mdpi.com/article/10.3390/ijms23105465/s1.
