# Supplementary material for: Identification and Transcriptome Analysis of Genes Related to Membrane Lipid Regulation in Sweet Sorghum under Salt Stress
Source: Int J Mol Sci. 2022 May 13;23(10):5465. doi: 10.3390/ijms23105465 (PMC9141458; doi:10.3390/ijms23105465)
Supplement: Supplementary file 1 [file ijms-23-05465-s001.zip › Table S1.pdf]

Table S1 List of primers for quantitative real-time PCR

Gene name	Primer sequence (5'-3')
SORBI_3010G001700	F: CCGCACTTTGGGCTGTACTA
	R: CTGTCGTCATATCCGGAGCC
SORBI_3004G286700	F: TTCTCGACACAACAGGGAGC
	R: TGACGTGGTTCTGGTACAGC
SORBI_3006G221500	F: TGTTCGGTCAGCTCTACGTG
	R: GGGAACAGGATGAGGGACAG
SORBI_3007G211900	F: TCGTGACGGATTTGGGGAAG
	R: TGATGATGATGTCCGGCTCG
SORBI_3003G150200	F: GTCTACCCCGAGATGTGGCA
	R: TGCCTTGAGCCAGTCGATGA
SORBI_3010G270700	F: GAGCGCCATCAGTACAGGAG
	R: ACAGTGGAATGTTGCCTCCC
SORBI_3003G318700	F: TGTGCCCAAGACAGATCCAC
	R: ACGCATAAGACACTTGGGCA
SORBI_3001G412100	F: CTGCCCACCGTGCTTACTAT
	R: CAGGCAGACTTGGGTCTCTG
SORBI_3001G365100	F: ACGCCAGCTCCAAGTACAAC
	R: GGCCTTGAAGAAAGGACCCC
SORBI_3010G160033	F: GGACTGGAAATGGCGGAAGA
	R: GTCAGGCAACCAGTCCATGA
SORBI_3001G448800	F: GCCAGGTCTTGAAGGTCGTT
	R: ACTCTCTCCTGCCTCCGATA
SORBI_3005G222500	F: GCGTGGAATGTGCAAGTGTT
	R: CAGGTGTCGGGTGACGTTTA
Actin	F: TGGCATCTCTCAGCACATTCC
	R: AATGGCTCTCTCGGCTTGC
